# Giant Craniopharyngioma Complicated by Cerebrospinal Fluid Ascites in a 7-Year-Old Boy: A Case Report and Review of Literature

**DOI:** 10.12669/pjms.41.13(PINS-NNOS).13507

**Published:** 2025-12

**Authors:** Maryem Tanweer, Syed Haider Hassan, Momin Bashir, Ahtesham Khizar

**Affiliations:** 1 Maryem Tanweer, MBBS, Postgraduate Resident Neurosurgery, Department of Neurosurgery Unit-I, Punjab Institute of Neurosciences, Lahore, Pakistan; 2 Syed Haider Hassan, MBBS, Research Intern, Department of Neurosurgery Unit-I, Punjab Institute of Neurosciences, Lahore, Pakistan; 3 Momin Bashir, Research Intern, Department of Neurosurgery Unit-I, Punjab Institute of Neurosciences, Lahore, Pakistan; 4 Ahtesham Khizar, MBBS, FCPS (Neurosurgery), Department of Neurosurgery Unit-I, Punjab Institute of Neurosciences, Lahore, Pakistan

**Keywords:** Craniopharyngioma, Ascites, Neurosurgery, Ventriculoperitoneal shunt, Pakistan

## Abstract

Craniopharyngioma (CPG) is a rare benign brain tumor often affecting the sellar/parasellar region in children. Giant variants can cause severe intracranial pressure and hydrocephalus and require ventriculoperitoneal (VP) shunting. A seven-year-old boy presented with a two-year history of headaches, as well as recent visual deterioration, altered sensorium, and vomiting. MRI brain revealed a cystic mass in the sellar and suprasellar regions causing mild hydrocephalus. After tumor excision and VP shunt placement, he developed abdominal CSF ascites, requiring shunt revisions, including ventriculoatrial shunt, and later suffered from a pseudomonas infection, leading to death from acute respiratory distress syndrome and hospital-acquired pneumonia. This case highlights the rare but critical complication of CSF ascites following VP shunting in a child with giant craniopharyngioma, emphasizing the importance of early recognition and timely intervention. Clinicians should be aware of this potential complication, as it requires careful diagnostic workup and management strategies, including shunt modifications and consideration of alternative shunting options.

## INTRODUCTION

Craniopharyngioma (CPG) is a rare benign intracranial tumor arising from residual epithelial cells of the craniopharynx, remnants of the Rathke’s pouch, or the epithelial rests of the embryonic craniopharyngeal duct.^1^ It is most commonly found in the sellar/parasellar region but can extend into the suprasellar area.^2^ CPG has a bimodal presentation, occurring most frequently in patients aged 5-14 years, followed by patients aged 50-74 years.^3^ There has been no gender difference observed in population studies, nor has any genetic underpinning been verified.^4^ CPG has an incidence rate of 0.13 cases per 100,000 per year, accounting for 2-4% of all intracranial neoplasia and 10% of pediatric brain tumors.^1^

Clinical symptoms of CPG include, but are not limited to, increased intracranial pressure, vomiting, headache, and visual impairment.^3^ The increased intracranial pressure can result in hydrocephalus, which poses a need for ventriculoperitoneal (VP) shunting.^5^ Surgical resection methods like microsurgery are an option available to treat this tumor, although they are associated with complications such as aggravated pituitary dysfunction, water-electrolyte imbalance, hypothalamic obesity, and neuropsychological disturbances.^6^ Adjuvant radiotherapy (RT) is considered an effective option to add to treatment, resulting in decreased postoperative morbidity.^6^ A rarer form of CPG is giant craniopharyngioma (GCPG). In adults, the mean size of CPG is approximately 3cm. In contrast, GCPG, defined as those exceeding 6cm in diameter or 60 mL in volume, are predominantly observed in the pediatric population.^3^

Similar to surgical resection, VP shunting, being an invasive procedure, carries risks of complications such as infection, intracranial hemorrhage, hematoma formation, and cerebrospinal fluid (CSF) ascites.^7^ In rare circumstances, GCPGs may be multicompartmental, producing severe intracranial pressure (ICP) and exacerbating hydrocephalus, thereby increasing the reliance on VP shunting and the likelihood of CSF ascites. Reporting such cases is important, as the coexistence of GCPGs with shunt-related CSF ascites is extremely rare, poses significant management challenges, and provides valuable insights into surgical decision-making and complication prevention in similar future cases. To our knowledge, this study reports the first such a complex case from Pakistan. We present the following case in accordance with the CARE guidelines.^8^

## CASE PRESENTATION

A 7-year-old boy presented to us in October 2023 with headaches from the last two years, left eye visual deterioration from the last month, altered sensorium, and vomiting for the last week. The general physical examination was normal, but on neurological examination he was found to have visual deterioration in the left eye with visual acuity of only light perception; the rest of the motor and sensory examination was normal.

**Fig.1:**
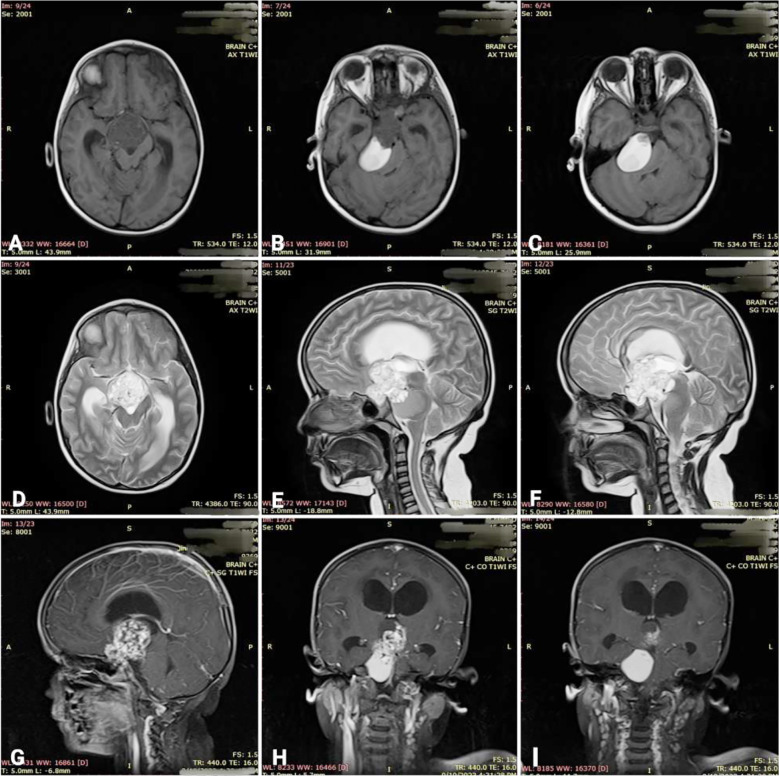
A, B & C: T1-weighted axial images showing a hypointense (solid) lesion in the sellar region and a hyperintense (cystic) infratentorial lesion. D: T2-weighted axial image showing hyperintense lesion. E & F: T2-weighted sagittal images showing the same lesion. G: T1-weighted contrast axial image showing heterogeneously enhancing lesion. H & F: T1-weighted contrast coronal images showing a heterogeneous lesion in the sellar/suprasellar region and a hyperintense infratentorial lesion.

On magnetic resonance imaging (MRI), a predominantly cystic complex lesion with extra-axial signal intensity of 5cm in anteroposterior diameter was seen, and an intrasellar mass was extending to the suprasellar area. The mass also had extensions into the interpeduncular cisterns and prepontine cisterns posteriorly and along the anterior surface of the right cerebellar hemisphere. On the T1-weighted image, there was high signal intensity suggesting internal hemorrhage or a cystic component with protein content; the mass effect of the tumor was also causing mild hydrocephalus. (Fig.1: A, B, C, D, E, F, G, H & I).

He was operated on for the tumor via right retrosigmoid craniectomy and excision of the right cerebellopontine angle (CPA) portion of the tumor, and external ventricular drainage (EVD) was placed. The retrosigmoid approach was opted for because the infratentorial portion of the lesion was causing significant brainstem compression. Moreover, the supratentorial portion was calcified, which is often not possible to remove. Postoperatively, EVD was changed to a ventriculoperitoneal (VP) shunt because of persistent hydrocephalus, and he got discharged one week after surgery at the same neurological status as preoperatively, on oral feed, and being mobile.

**Fig.2:**
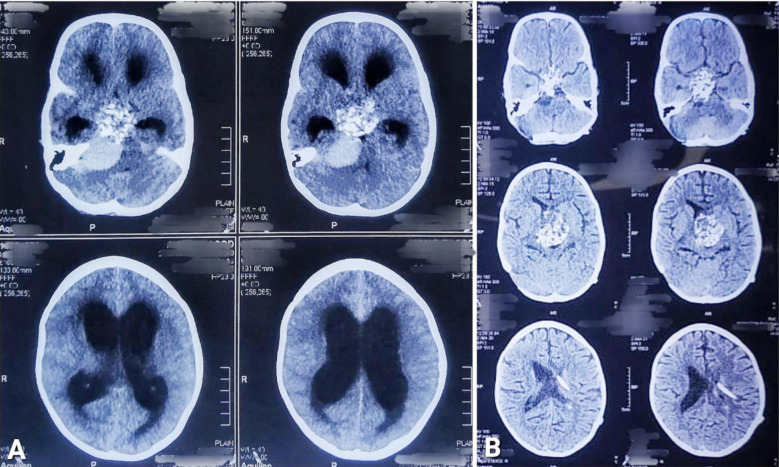
A: A CT brain plain showing a hyperdense sellar/suprasellar lesion with a hyperdense infratentorial CPA lesion and ventriculomegaly suggestive of hydrocephalus. B: Postoperative CT brain plain showing presence of sellar/suprasellar lesion, right retrosigmoid suboccipital craniectomy defect, and in situ VP shunt on left side.

**Fig.3:**
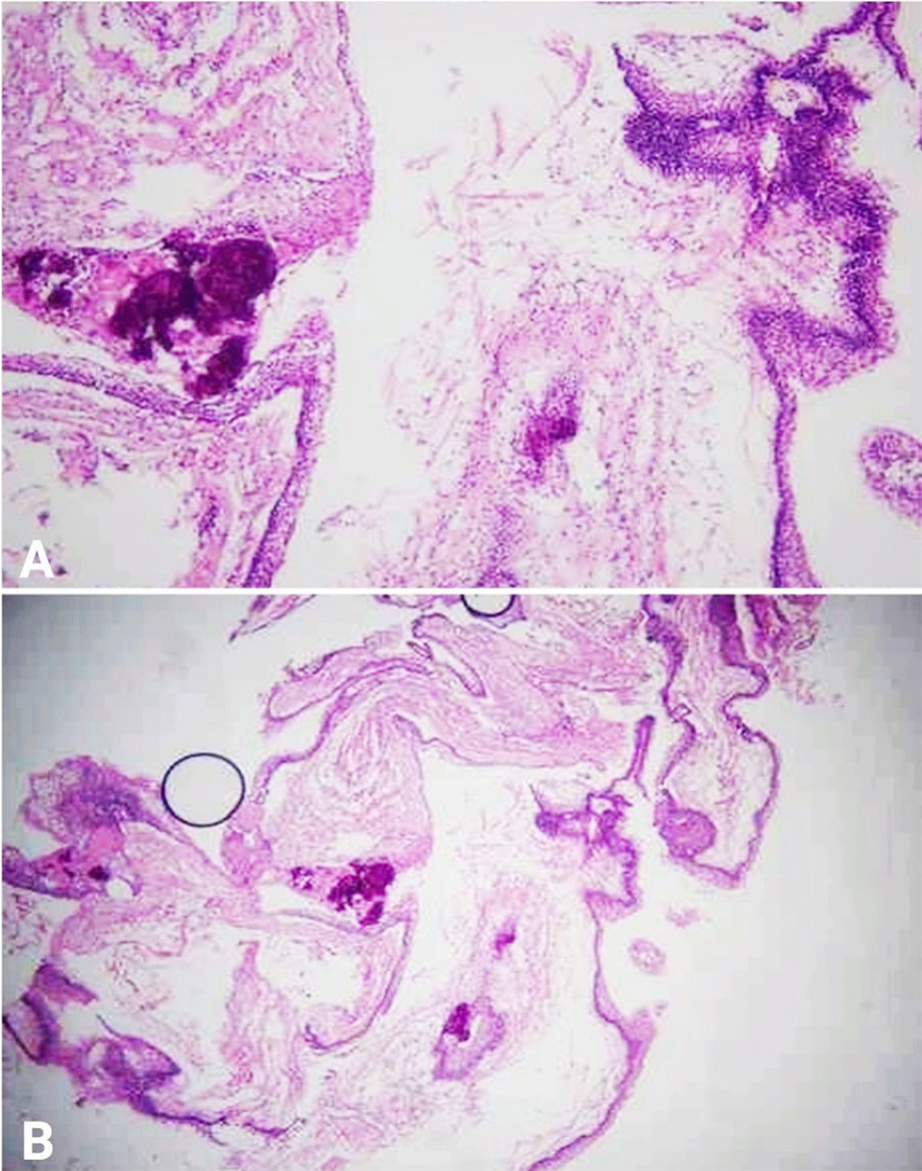
A & B: Histological examination of the section revealed multiple fragments. Few of the fragments showed squamous lining and stellate reticulum. Areas of calcification, reverse polarity, wet keratin, and ameloblast were also seen. A diagnosis of WHO Grade-I adamantinomatous CPG was made.

Preoperative and postoperative computed tomography (CT) brain scans along with findings are shown in Fig.2: A & B. One month after his surgery, he again presented with headache and vomiting in the emergency department; a brain CT showed hydrocephalus, for which VP shunt lower end revision was required, and the patient was discharged with a working shunt and resolved symptoms. By that time, the tumor biopsy report came out as WHO Grade-I adamantinomatous CPG (Fig.3: A & B), for which the patient was referred to the neuro-oncology department, and they planned radiosurgery of the residual tumor, but it could not be done as the tumor was bulky enough (46 x 37 x 49 mm suprasellar mass extending to intrasellar and right CPA with 13 x 9 mm cystic component), and the optic chiasm was encased as well.

**Fig.4:**
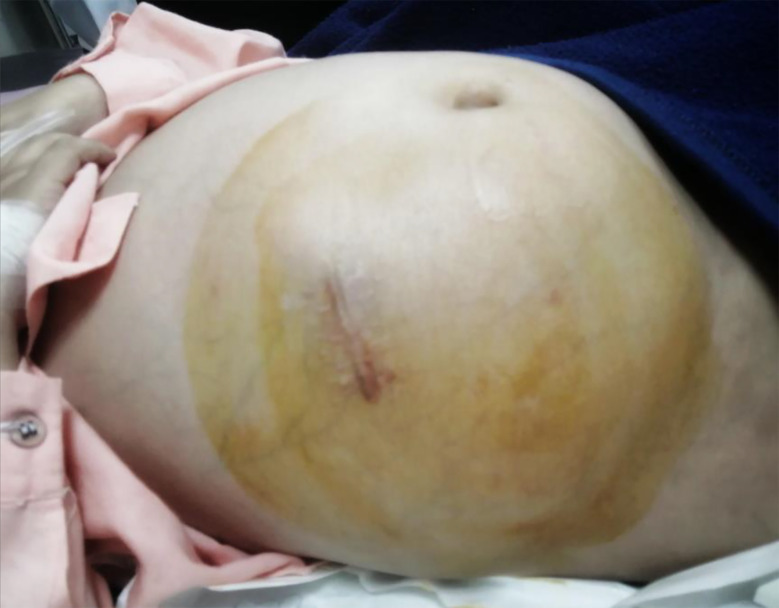
Abdominal distension due to CSF ascites.

The patient was fine for one month with no progression of symptoms when he again presented with multiple episodes of vomiting and abdominal distension (Fig.4) for one week; abdominal distension was gradually increasing and associated with constipation. Neurological examination showed bilateral decreased visual acuity to light perception; the VP shunt was working with a good refill of the chamber. Baseline investigations were normal. Ultrasound (USG) of the abdomen showed free fluid in the abdomen with normal liver and kidney structures, ruling out any liver or kidney pathology.

A diagnostic and therapeutic abdominal tap was performed, and approximately 540 ml of abdominal fluid was drained. On fluid analysis, CSF ascites was diagnosed, for which the lower end of the VP shunt was exteriorized along with abdominal paracentesis (250 ml of ascitic fluid was removed this time). The abdominal ascitic fluid cytology report showed glucose 103 mg/dl, protein 0.8 g/dl, and LDH 123 U/l. Absence of any malignant cells ruled out malignant ascites.

Abdominal fluid cytology and resolution of abdominal distention and vomiting after shunt lower-end exteriorization confirmed the diagnosis of abdominal CSF ascites, and we planned to do a ventriculoatrial (VA) shunt. The patient was on the list for a VA shunt when he suddenly deteriorated to GCS 12 with the VP shunt chamber collapsed, and an EVD from the right Kocher’s point was done at night, followed by a VA shunt in the morning, and the removal of the EVD was done. The patient got discharged later on after the VA shunt with GCS 15 and on NG feed.

In March 2024, the patient again presented with a history of high-grade fever and two episodes of generalized tonic-clonic seizures for one day. The patient was admitted indoors at GCS 15, and blood, urine, and CSF samples (taken via ventricular tap from a previous burr hole done for EVD) were sent for complete examination and cultures, while ceftriaxone and vancomycin were started after consulting with a pediatrician. The patient’s GCS remained 15, but his fever was not responding to any medications started empirically. His WBC count on a complete blood examination was 37000, ESR 72, and CRP 32. CSF analysis showed TLC of 130/cmm.

Urine and blood culture reports were normal, while the CSF culture report showed moderate growth of pseudomonas species, for which antibiotics according to sensitivity were started. Fever intensity started reducing after that, with a fever documented of 100°F on and off. But the patient’s condition deteriorated again in one month’s time; his GCS dropped to seven with the shunt chamber collapsed, and EVD from the previous burr hole on the right Kocher’s point was done again, and the patient was put on a ventilator in SIMV mode. VA shunt upper end revision and removal of EVD were done subsequently once a complete CSF examination showed a TLC count <5/cmm. As the CSF analysis was clear, culture was not performed at this time.

Although the VA shunt was working, GCS remained static at 7T, but the patient could not be weaned off from the ventilator, for which the pulmonology department was consulted, and they started management on the line of acute respiratory distress syndrome due to hospital-acquired pneumonia. The patient did not show much response to medical treatment, with the condition worsening gradually until he expired in May 2024.

## DISCUSSION

One rare complication of VP shunting is CSF ascites, which may be the result of either excessive production of CSF or the inability of the peritoneum to absorb it. Choroid plexus hyperplasia causes increased production of CSF, while peritoneal malabsorption stems from peritoneal irritation due to recent surgery, shunt material allergy, or high CSF protein as found in CPG or optic pathway gliomas.^9^ Another pathophysiological mechanism underlying optic glioma-related CSF ascites is the defect in the blood-brain barrier causing leakage of tumor proteins into the subarachnoid space and hence increasing protein levels.^10^ A study by Trevisi et al. and Hori et al. mentioned choroid plexus hyperplasia as the cause of excessive CSF production.^11^ But, in our present case, imaging rules out the possibility of choroid plexus hyperplasia. In 2016, Jamal et al. proposed that increased concentrations of proteins in CSF from tumors, infection, or inflammation of any type lead to high intraperitoneal oncotic pressure and eventually impaired peritoneal CSF absorption.^12^ But the abdominal fluid analysis of our patient showed a protein level of 0.8 g/dL, thus making this mechanism less likely but not completely excluding it, as CPG diagnosed on histopathological examination of this patient led to increased ICP. And the greater the increase in ICP, the greater the need for VP shunting and the greater the risk of the development of CSF ascites. Although CSF protein levels were low, the development of ascites may be more likely attributable to a reduced capacity of the peritoneum to adequately absorb CSF.

Among the reported cases of CSF ascites, 10 were ≥18 years old, and one was over 65 years old.^13^ Also, no gender predominance was seen. Our case is unique in the way that the child was a seven years old boy who was diagnosed with GCPG and developed CSF ascites post-VP shunting. The interval between placement of the VP shunt and the development of ascites varies between one and 12 years.^14^ In this case presentation, it was observed eight weeks after the initial VP shunt.

The presenting complaint in this boy was intractable vomiting and abdominal distension for one week. The abdominal distension was progressive, and it was also associated with constipation. Wu et al. also reported massive abdominal distension, bilateral pleural effusion, and bilateral hydrocele occurring concurrently in an 89-year-old man.^13^ The likely explanation in the latter case is the presence of micro-communications between the peritoneal and pleural cavities and the peritoneum and scrotum, respectively.

The proposed treatment with a high success rate is VP to VA shunt conversion.^7^ Also, placing a Denver shunt has also shown promising results.^15^ In this 7-year-old boy who presented with abdominal distension post-shunt placement, initially exteriorization of the lower end of the VP shunt and abdominal paracentesis were done, followed by VA shunt, resulting in resolution of vomiting and abdominal distension. There was a slight delay in VA shunting owing to the deterioration of the patient’s GCS to 12/15 and collapse of the VP shunt chamber, for which EVD was done on an emergency basis, followed by VA shunting in the morning. The rationale for paracentesis was to provide diagnostic and therapeutic yield, and it was helpful in reducing the patient’s abdominal pain and distension.

To establish the diagnosis of CSF ascites, it is important to rule out the most common etiologies, namely, liver cirrhosis, heart failure, and nephrotic syndrome.^13^ In our case, liver and kidney pathology was ruled out on the basis of normal LFTs, RFTs, serum electrolytes, and normal hepatic and kidney structures on ultrasonography. Heart failure seemed less likely as the clinical signs of dyspnea, lower extremity edema, and hepatojugular reflex were absent. B2-transferrin is a biomarker with almost 99% specificity for CSF,^16^ but it was not utilized in this case due to non-availability, and hence the resolution of abdominal distention and vomiting after shunt lower-end exteriorization confirmed the diagnosis of abdominal CSF ascites.

The rate of infections seen with CSF catheters ranges from 5 to 47%. Most of the contaminations occur with skin flora at the time of surgery or may be related to spreading from distant sites of infection.^17^ Staphylococcus is the most likely implicated organism, followed by E. coli.^9^ In this case also, when the patient presented 12 days post-VA shunting with high-grade fever and seizures, pseudomonas was isolated from CSF. This organism was most likely hospital acquired owing to the rapidity and frequency with which multiple EVDs and shunt replacements were done in the patient due to this fluctuating GCS. It was the severity of this shunt complication that the patient eventually succumbed to.

CPGs are broadly classified into adamantinomatous and papillary types. Adamantinomatous occurs predominantly in children, while the papillary type is more common in adults.^18^ Likewise, this child also turned out to have the adamantinomatous type. BRAF V600E mutations were detected in 95% of PCP patients, while 75-96% of ACP patients had CTNNB1 mutations.^19^ Genetic testing for these mutations was not done in our case due to lack of availability.

A detailed literature review on GCPG is given in Table-I. The literature suggests that the patients diagnosed with GCPG usually have a mean age of 25 years. However, three cases have been reported to be of 4, 10, and 13 years, respectively. Likewise, our seven years old boy has an atypical presenting age of a GCPG.

**Table-I:** Details of literature review on giant craniopharyngiomas.

Authors	Article Topic	Age (years)	Gender	Site	Size (cm)	Management
Chen et al.^1^	Neuro-endoscopic treatment of GCPG in the foramen magnum: report of 2 cases	i. 10	Male	Giant cystic lesion on the right middle and posterior fossa.	-	Micro-endoscopic excision of cystic CPG via a 1*1.5 cm hole in the lateral margin of the right orbit. No postoperative complications. Followed for 3 years; no tumor recurrence was noticed.
		ii. 15	Male	Giant cystic lesion in the frontal, middle, and posterior fossa also involving the third ventricle.	-	Micro-endoscopic excision of cystic tumor via right frontal along with Ommaya reservoir. Follow-up after 5 years: VP shunt done for recurrent tumor on remote site (solid+cystic) causing hydrocephalus, and discharged with relieved symptoms. Again, after 1 year, neuro-endoscopic excision was done for an enlarged recurrent tumor (cystic portion only). The residual solid portion did not grow in size in the follow-up of 2 years.
Luo et al.^3^	A case of giant cystic adamantinomatous CPG in an adult	27	Male	Anterior pontine and superior sella turcica cistern.	5.2*2.6*6.1	Surgical excision of the tumor.
Sadhasivam et al.^6^	The implication of giant tumor size on surgical resection, oncological, and functional outcomes in CPG	Total 44 patients: 25 patients (<18y), 16 patients (>18y)	-	-	>4cm	17 patients had gross total resection. 27 had subtotal resection. Of them, 11 patients had adjuvant therapy.
Saluja et al.^26^	See-saw nystagmus in GCPG	4	Male	Suprasellar solid and cystic, causing compression of the third ventricle.	-	-
Tian et al.^2^	CPG involving the anterior, middle, and posterior fossa in adults: A case report	46	Male	Multifocal cystic masses in the anterior, middle, and posterior fossa.	-	Surgical excision of tumor in 2 sittings: 1st resection of middle and posterior fossa tumor via right retrosigmoid sinus approach, followed by 2nd surgery after 4 months via subfrontal interhemispheric approach for anterior fossa tumor.
Jha et al.^5^	Complications and outcome in GCPG: A case series and meta-analysis	i. 13 ii. 24 iii. 19 iv. 18 v. 70	Male	Multi-compartmental extension, including clival and subtemporal extension.	-	Complete excision in 2 patients, subtotal resection in 2, and decompression in the other 2 patients.

There is an overwhelmingly male predominance, as was seen in this case as well. The review also highlights that CPG primarily grows along the midline, particularly in the sella turcica, with some degree of suprasellar involvement as well. Additionally, it can present simultaneously in the sellar and suprasellar regions.^20^ A few cases have been reported to occur in the lateral ventricle, 3^rd^ ventricle, and even the nasopharynx.^21^ These all locations are likely explained by the path of migration of Rathke’s sac, so tumorigenesis can occur anywhere along the pathway.

In this case report, we describe a child with an intrasellar mass extending to the suprasellar area. The mass also had extensions into the interpeduncular cisterns and prepontine cisterns posteriorly and along the anterior surface of the right cerebellar hemisphere. Both the size (≥5 cm in anteroposterior diameter) and the extent of the tumor being multi-compartmental, which is rarely seen in the scholarly articles, pointed to the possibility of a GCPG. Also, the high signal intensity seen on T1 suggested internal hemorrhage or a cystic component with some protein. The literature also supports the fact that high T1 signal intensity is due to high cholesterol or protein levels, mild calcification, and bleeding.^22^

The optimal treatment is total surgical resection, but in the case of challenging resection, like in this case, it is advocated to utilize adjuvant therapies like radiotherapy, chemotherapy, and targeted therapy or palliative therapies like vesicular aspiration.^23^ The approaches for surgical entry are varied and include the pterional approach, staged procedures through pterional and suboccipital approaches, and the Kawase approach.^24^ A study by Schubert et al. demonstrated endoscopic drainage of cystic CPG had better endocrine outcomes than microsurgery.^25^ In the aforementioned case, adjuvant radiotherapy was an option, but owing to the tumor bulk (46 x 37 x 49 mm) and encasement of the optic chiasm, radiotherapy was deferred. Though the residual lesion measured 4.9 cm; however, it consisted of a densely calcified component, which is often technically challenging or unfeasible to excise completely. Beyond this residual mass in our case, the clinical course was further complicated by shunt-related issues, most notably CSF ascites, which significantly affected management.

## CONCLUSION

It is important to note that the standard method of treating craniopharyngioma is complete resection, but it is challenging in the case of giant craniopharyngioma. However, endoscopy, which removes as much of the tumor as possible, and adjuvant radiotherapy can yield satisfying results. In the case of CSF ascites, it is vital to elucidate the underlying cause, as it will dictate the treatment. It might be essential to remove the shunt in case of infections or may need switching to a ventriculo-atrial shunt in case of tumors.

### Abbreviations:

**CPA:** Cerebellopontine angle; **CSF:** Cerebrospinal fluid; **CPG:** Craniopharyngioma; **CT:** Computed tomography; **EVD:** External ventricular drain; **GCPG:** Giant craniopharyngioma; **GCS:** Glasgow coma scale; **ICP:** Intracranial pressure; **MRI:** Magnetic resonance imaging; **RT:** Radiotherapy; **SRS:** Stereotactic radiosurgery; **USG:** Ultrasound; **VA:** Ventriculoatrial; **VP:** Ventriculoperitoneal.

### Authors’ Contributions:

**MT, SHH, MB:** Data acquisition, analysis, interpretation, drafted the manuscript.

**AK:** Concept and design of study, critical review of the manuscript and supervision.

All the authors have read and approved the final manuscript for the publication. All agreed to be accountable for all aspects of the work related to accuracy or integrity.

## References

[1] Chen A, Zhou R, Yao X, Ai M, Sun T (2021). Neuroendoscopic treatment of giant cystic craniopharyngioma in the foramen magnum:report of two cases. Childs Nerv Syst.

[2] Tian B, Li M, Du X, Zhou H, Zhou K, Li S (2023). Craniopharyngioma involving the anterior, middle, and posterior cranial fossa in adults:A case report. Front Neurol.

[3] Luo SP, Zhang HW, Yu J, Jiao J, Yang J, Lei Y (2020). A rare case of giant cystic adamantinomatous craniopharyngioma in an adult. Radiol Case Rep.

[4] Müller HL (2014). Craniopharyngioma. Endocr Rev.

[5] Jha RK, Deepak K (2024). Complications and outcome in giant craniopharyngioma-a case series and meta-analysis. J Cardiovasc Dis Res.

[6] Sadhasivam S, Menon G, Abraham M, Nair SN (2020). The implication of giant tumor size on surgical resection, oncological, and functional outcomes in craniopharyngioma. Pituitary.

[7] Khizar A, Zahid S (2022). Delayed cerebrospinal fluid ascites following ventriculoperitoneal shunt:A case report with literature review. Rom Neurosurg.

[8] Riley DS, Barber MS, Kienle GS, Aronson JK, Von Schoen-Angerer T, Tugwell P (2017). CARE guidelines for case reports:explanation and elaboration document. J Clin Epidemiol.

[9] Alraee S, Alshowmer S, Alnamshan M, Azzubi M (2020). Management of ventriculo-gallbladder shunt in the presence of gallstones. BMJ Case Rep.

[10] Tang TT, Whelan HT, Meyer GA, Strother DR, Blank EL, Camitta BM (1991). Optic chiasm glioma associated with inappropriate secretion of antidiuretic hormone, cerebral ischemia, nonobstructive hydrocephalus and chronic ascites following ventriculoperitoneal shunting. Childs Nerv Syst.

[11] Trevisi G, Frassanito P, Di Rocco C (2014). Idiopathic cerebrospinal fluid overproduction:case-based review of the pathophysiological mechanism implied in the cerebrospinal fluid production. Croat Med J.

[12] Jamal H, Abrams G (2016). A corny cause of cerebrospinal fluid ascites:A case report and review of literature. SAGE Open Med Case Rep.

[13] Wu X, Sandhu M, Dhand R, Alkukhun L, Lamichhane J (2021). Sterile cerebrospinal fluid ascites, hydrothorax and hydrocele as a complication of ventriculoperitoneal shunting in an elderly patient. BMJ Case Rep.

[14] Nwang'ombe NJ, Thiong'o GM, Boore JK (2021). Cerebrospinal fluid ascites. A case report and literature review. African J Neurol Sci.

[15] Musa G, Gots A, Lungu MC, Mutumwa M, Mutumwa M (2019). Cerebrospinal fluid ascites:A patient case report and literature review. Med J Zambia.

[16] Warnecke A, Averbeck T, Wurster U, Harmening M, Lenarz T, Stöver T (2004). Diagnostic relevance of beta2-transferrin for the detection of cerebrospinal fluid fistulas. Arch Otolaryngol Head Neck Surg.

[17] Wallace AN, McConathy J, Menias CO, Bhalla S, Wippold II FJ (2013). Imaging evaluation of CSF shunts. AJR Am J Roentgenol.

[18] Lee IH, Zan E, Bell WR, Burger PC, Sung H, Yousem DM (2016). Craniopharyngiomas:radiological differentiation of two types. J Korean Neurosurg Soc.

[19] Chen X, Tong Y, Shi Z, Chen H, Yang Z, Wang Y (2019). Noninvasive molecular diagnosis of craniopharyngioma with MRI-based radiomics approach. BMC Neurol.

[20] Mahdi MA, Krauss JK, Nakamura M, Brandis A, Hong B (2018). Early ectopic recurrence of craniopharyngioma in the cerebellopontine angle. Turk Neurosurg.

[21] Palmer JD, Song A, Shi W, Chang E, Brown P, Lo S, Sahgal A, Suh J Craniopharyngioma. Adult CNS Radiation Oncology.

[22] D'Anna G, Grimaldi M, Scotti G (2016). Neuroradiological diagnosis of craniopharyngiomas. Diagn Manage Craniopharyng.

[23] Grob S, Mirsky DM, Donson AM, Dahl N, Foreman NK, Hoffman LN (2019). Targeting IL-6 is a potential treatment for primary cystic craniopharyngioma. Front Oncol.

[24] Kiran NA, Suri A, Kasliwal MK, Garg A, Ahmad FU, Mahapatra AK (2008). Gross total excision of pediatric giant cystic craniopharyngioma with huge retroclival extension to the level of foramen magnum by anterior trans petrous approach:report of two cases and review of literature. Childs Nerv Syst.

[25] Schubert T, Trippel M, Tacke U, Van Velthoven V, Gumpp V, Bartelt S (2009). Neurosurgical treatment strategies in childhood craniopharyngiomas:is less more?. Childs Nerv Syst.

[26] Saluja G, Samdani A, Bhatia P (2020). See-saw nystagmus in giant craniopharyngioma. BMJ Case Rep.

